# Finding Common Ground: Indigenous Research Methods Facilitate Scientific Knowledge Sharing in Cross‐Cultural Wildlife Research

**DOI:** 10.1002/ece3.72274

**Published:** 2025-10-10

**Authors:** Bridget Campbell, Banygada Brendan Wunuŋmurra, Butjiyaŋanybuy Thomas Marrkula, Munurruŋ Bobby Wunuŋmurra, Yirralka Rangers, Frances Morphy, Emilie Ens

**Affiliations:** ^1^ School of Natural Sciences Macquarie University Sydney New South Wales Australia; ^2^ Laynhapuy Homelands Aboriginal Corporation Yirrkala Northern Territory Australia; ^3^ Centre for Indigenous Policy Research Australian National University Canberra Australian Capital Territory Australia

**Keywords:** biocultural conservation, both‐ways ecology, mutual incomprehension, science communication, traditional ecological knowledge, Yolŋu

## Abstract

Western scientists, when collaborating with Indigenous Peoples in conservation science, tend to assume mutual comprehension between parties, including of concepts, knowledge systems, priorities and communication of results. Failure to acknowledge the possibility of mutual incomprehension is likely an artefact of Western science hegemony, resulting in assumptions that Western concepts are universally understood and accepted. Deliberate actions to ensure mutual understanding are essential if we are to decolonise conservation science towards more equitable collaborations. In the present viewpoint, our cross‐cultural research team of Yolŋu (Indigenous) and Balanda (Western, here Euro‐ Australian) researchers reflect on the application of Yolŋu dhawurrpunaramirri (both‐ways discussion and negotiation) as an Indigenous research method to guide the cross‐cultural negotiation of Western science concepts and results. These concepts and results were central to a wildlife genetics research project conducted collaboratively by the research team, published as a part of this special issue (Campbell et al. in review). Through dhawurrpunaramirri we identified and negotiated key Yolŋu Matha (language) terms that enabled discussion and clarification of Western scientific ontology and epistemology. Yolŋu social organisation (including clan‐based and ceremonial connections) emerged as an overarching source of metaphor to culturally inform mutual comprehension of genetics concepts, research results and to generate communication outputs. The Yolŋu terms miṯtji, mala and bäpurru were discussed to contextualise and explain the Balanda notion of ‘species’. The Yolŋu concept of gurrkurr (venous system, and by metaphorical extension, root system and ‘blood line’) was suggested as meaningful to contextualise phylogenetic trees. Related Western genetics concepts of speciation, phylogenetics, DNA, scientist and sequence were also discussed, negotiating and contrasting meanings from Balanda and Yolŋu scientific knowledge systems, whilst balancing ontological incommensurability. We recommend other researchers and cross‐cultural research teams engage in Indigenous research methods to identify and improve mutual comprehension and enhance local understanding of genetics research (here; Campbell et al. in review). By finding common ground, respecting Indigenous knowledges and negotiating meanings across knowledge systems we work towards decolonising science and advancing global biocultural conservation and Indigenous rights.

## Graphical abstract

Indigenous research methods can be applied in cross‐cultural conservation research to investigate and remedy mutual incomprehension. Here, through the application of Yolŋu dhawurrpunaramirri (both‐ways discussion and negotiation), we (Yolŋu and Balanda co‐researchers) identified Yolŋu Matha (language) concepts and metaphors derived from characteristics of Yolŋu social organisation and relationality that helped contextualise and explain Western scientific concepts and fauna genetics results. Generating a ‘common ground’ of mutual understanding in a culturally grounded way, facilitated more equitable knowledge sharing. In doing so we attempted to decolonise scientific practice and highlight Yolŋu ways of understanding and relating to the world.

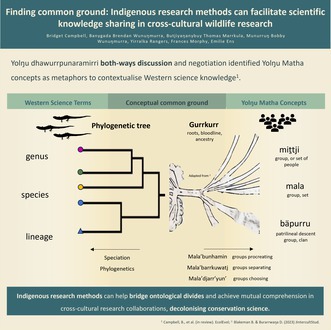

## 1 Introduction

International calls for cross‐cultural collaborations between Western scientists and Indigenous Peoples in conservation and research are increasing (Berkes 1999; IPBES 2019; Tengö et al. 2017). These calls seek to address interdependent losses of biological and cultural diversity, situating Indigenous Peoples and Indigenous knowledge as central to solutions (Ens et al. 2021; Garnett et al. 2018; IPBES 2019; UNESCO‐SCBD 2016). Despite this, in the cross‐cultural conservation literature, little explicit attention is given to the fundamental and likely pervasive underlying challenge of mutual incomprehension that is widely recognised in other fields such as the health and education sectors (Maddox et al. 2022; Sanga et al. 2018; Sharmil et al. 2021).

Mutual comprehension is defined as the direct exchange and understanding of ideas and terms between people of different cultures and languages (Lin and Liu 2012). As a contemporary global problem, the converse, mutual incomprehension, remains a key area of research to improve intercultural understanding, interactions and communication in the fields of linguistics and psychology (Keysar et al. 2000; Lin et al. 2015; Lin and Liu 2012) with increasing application in the education and health sectors (Affonso et al. 1996; Bishop 2012; Maddox et al. 2022; Sanga et al. 2018; Sharmil et al. 2021). Mutual incomprehension has been recognised as a core challenge in cross‐cultural contexts, particularly where colonial systems have delegitimised Indigenous epistemologies and communicative norms (McRae‐Williams and Gerritsen 2010). Similarly, Western research methods, ‘embedded in a global system of imperialism and power’ (p286), are increasingly criticised for unethical, hegemonic relationships of exploitation and misrepresentation, especially with Indigenous Peoples (Smith 2012).

In the conservation sector, mutual incomprehension between Western and Indigenous actors has contributed to strong Indigenous advocacy for recognition and inclusion of Indigenous knowledge, values, priorities and language in conservation research and decision making (Smith 2012; Collier‐Robinson et al. 2019; Goolmeer et al. 2022) and imperatives to decolonise science (Howitt and Suchet‐Pearson 2003, 2006; Moon and Pérez‐Hämmerle 2022). Here, our cross‐cultural research team of Yolŋu (Indigenous) and Balanda (Western, here Euro‐ Australian) researchers reflect on the application of Yolŋu dhawurrpunaramirri (both‐ways discussion and negotiation) to guide the cross‐cultural negotiation of Western science concepts and genetics research results in an attempt to decolonise Western scientific communications.

Investigations of mutual incomprehension in cross‐cultural research are usually pursued through the application of qualitative, social science methods (McRae‐Williams and Gerritsen 2010) and when multiple languages are involved, linguistics (Lin and Liu 2012). Despite the growing recognition of cross‐disciplinary methods, most Western scientists are often not trained in multidisciplinary, social science or linguistic research methods. We suggest that this disciplinary or academic divide between the environmental and social sciences (alongside the hegemony of Western science) has allowed mutual incomprehension to go undetected in conservation science and perpetuated invisible barriers to equitable knowledge sharing and decision making (Beck et al. 2021; Reed et al. 2024). Explicit attempts to recognise and bridge mutual incomprehension in real‐world conservation projects are rare but beginning to emerge with the increasing promotion of Indigenous research methods in cross‐cultural conservation pursuits (Beveridge et al. 2021; Reed et al. 2024). Here we reflect on our detection of mutual incomprehension in a cross‐cultural wildlife genetics research project and through application of Yolŋu dhawurrpunaramirri (both‐ways discussion and negotiation) (Wunuŋmurra 1989) as an Indigenous research methodology. We used this method to negotiate mutual comprehension of Western scientific genetics results by discussing and negotiating linguistic translations of the following key concepts: species, phylogenetic tree, speciation and phylogenetics.

## 2 Decolonising Science: Indigenous Research Methods

Indigenous research methods have only recently emerged in the academic literature. They offer culturally grounded alternatives that counter the ongoing colonial practices of Western research imposed on Indigenous Peoples (Kovach 2017; Smith 2012). Indigenous research methods privilege Indigenous ontologies, relationality and ways of knowing, being and doing, supporting Indigenous rights and self‐determination (Beveridge et al. 2021; Geia et al. 2013; Kovach 2017). The preferred methods vary by context, aligning with local practice and protocol. However, they are generally centred around: cultural accountability; responsible, contextualised approaches; reflexivity and respectful representation of Indigenous peoples, knowledge and Country (Beveridge et al. 2021; Kovach 2017). Most Indigenous research methods emphasise the adoption of conversational styles and/or narrative approaches, for example, by Aboriginal Australians (Bessarab and Ng'andu 2010; Ungunmerr‐Baumann et al. 2022), Pasifika Peoples (Sanga et al. 2018), Kānaka Maoli (Native Hawaiians; Au and Kawakami 1985) and Canadian First Nations Peoples (Kovach 2010). In contrast to most Western qualitative research methods, Indigenous research methods are not unidirectional but are reciprocal and mutual and involve relationship building and researcher accountability (Bessarab and Ng'andu 2010; Geia et al. 2013; Maddox et al. 2022).

Indigenous and non‐Indigenous researchers have also applied Indigenous research methods in concert with Western approaches, adopting two‐way or cross‐cultural methods. For example, Sharmil et al. (2021) combined participatory action research and Yarning approaches, Kahakalau (2004) combined heuristic approaches and talk story and Ens and colleagues combined quantitative (ecological) and qualitative (‘Yarning‐style’ interview) approaches (Ens et al. 2016; Sloane et al. 2019; Russell et al. 2020; Daniels et al. 2022; McKemey et al. 2022; Campbell et al. 2022, Campbell et al. 2024). Two‐way and Indigenous research applications generally draw on Indigenous knowledge and priorities to guide overall project and methodological (monitoring/sampling) development (see Beveridge et al. 2021; Collier‐Robinson et al. 2019; Goolmeer et al. 2024; Rayne et al. 2022; Skroblin et al. 2022).

## 3 Engaging With Local Indigenous Research Methods: Dhawurrpunaramirri

In north east Arnhem Land, Australia, dhawurrpunaramirri (lit: interrupting each other) was proposed by Wunuŋmurra (1989) as an approach to negotiating Yolŋu and Balanda knowledge in the generation of a ‘two‐way’ bilingual curriculum for the Yirrkala school. Dhawurrpunaramirri is an Indigenous, culturally grounded approach that is not unidirectional, but requires bala ga lili (back and forth) exchange and negotiation of meanings from participants. The aim of such negotiation is to generate ‘common ground’ or mutual comprehension between Yolŋu and Balanda to allow for respectful and equitable decision making and allow Yolŋu to live in both worlds, without ‘destruction’ of their culture (Marika et al. 2009; Muller 2014; van Gelderen and Guthadjaka 2021; Wunuŋmurra 1989). Dhawurrpunaramirri is about people coming together from different places to respectfully navigate the barriers of mutual incomprehension, and overcoming them in situ by creating, sharing and negotiating knowledges (Wunuŋmurra 1989).

**FIGURE 1:**
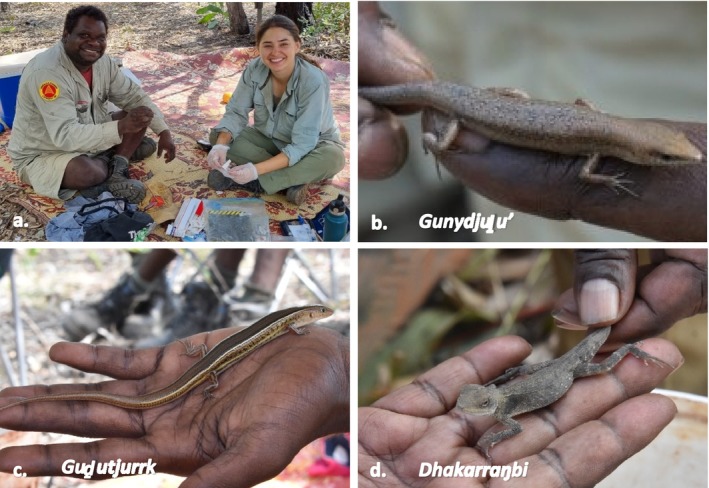
(a) Authors BBW and BC collecting tissue samples from (b) gunydjuḻu (small skinks, including *Carlia* spp), (c) guḏutjurrk (large skinks, including *Ctenotus* spp) and (d) dhakarraŋbi (small dragons, including *Diporiphora* spp) at a cross‐cultural survey camp at Garrata in 2022. These lizards were the source of mutual incomprehension during surveys as Yolŋu classifications and Western taxonomies did not map directly onto each other. Yolŋu classifications of gunydjuḻu, guḏutjurrk, dhakarraŋbi contain many distinct genera and species in Western taxonomy.

Here we adopted Yolŋu both‐ways dhawurrpunaramirri negotiation as a locally relevant approach to attain mutual comprehension of the results of a wildlife genetics project. This project was a collaboration between the Yolŋu Yirralka Rangers and Balanda university researchers, reported in Campbell et al. (*in review*) and Campbell (2025), that applied genetic tools to investigate lizard diversity in east Arnhem Land, northern Australia. These genetic tools were used to determine: (1) species (and lineage) identification; (2) species distribution; and (3) population structure of three speciose genera of small lizards, *Carlia* and *Ctenotus* skinks and *Diporiphora* dragons. In Yolŋu Matha, these lizards fall under the categories of gunydjuḻu, guḏutjurrk and dhakarraŋbi (Figure 1). From a Western science perspective, genetic tools were used to resolve species‐level field identifications of *Carlia, Ctenotus andDiporiphora spp*. These lizards were difficult to identify in the field due to subtle differences in morphological characteristics, in part due to morphologically cryptic species complexes and recent taxonomic revisions based on genetic markers (Fenker et al. 2021; Melville et al. 2019; Prates et al. 2022, 2023). The methods and results of these analyses were subsequently negotiated through dhawurrpunaramirri to develop mutual understanding of species boundaries in relation to Yolŋu classifications.

## 4 Recognising Mutual Incomprehension

Discussions between the Yolŋu Yirralka Rangers and Balanda university ecologists about the genetics research illuminated mutual incomprehension around animal classifications and Western scientific concepts. For Yolŋu researchers, the lizards of interest included three types of Yolŋu‐recognised animals: gunydjuḻu (small skinks), guḏutjurrk (large two‐lined skinks) and dhakarraŋbi (small dragons) (Figure 1). For Balanda researchers, there existed multiple species within three key genera: *Carlia* (rainbow skinks), *Ctenotus* (comb‐eared skinks) and *Diporiphora* (two‐lined dragons). A key part of the broader cross‐cultural fauna survey collaboration was to identify and record fauna observations using Yolŋu and Balanda nomenclature while negotiating meanings across the two systems as part of the research process. However, it became apparent that Balanda researchers were attempting to ‘identify’ fauna in a way not recognised by Yolŋu, using methods (genetic analysis) and reasoning (the species concept) founded on a way of understanding and relating to the world (ontology and epistemology) that was foreign to Yolŋu (Campbell et al. 2024).

## 5 Dhawurrpunaramirri Approach: Resolving Mutual Incomprehension

In the present viewpoint, we share how we, as Balanda and Yolŋu co‐researchers, engaged in dhawurrpunaramirri to explain fauna genetics results using Western and Yolŋu concepts to enable mutual comprehension. Authors BBW, BTM, MBW (Yolŋu Yirralka Rangers) and BC (Macquarie University cross‐cultural ecology researcher) engaged in dhawurrpunaramirri negotiation with each other, five Ŋaḻapaḻmi (senior Yolŋu knowledge holders) and four Yirralka Rangers. Authors BBW, BTM, MBW and BC are privileged with comfortable, trusting working relationships, having worked together for five years on a range of cross‐cultural ecology research projects.

**FIGURE 2:**
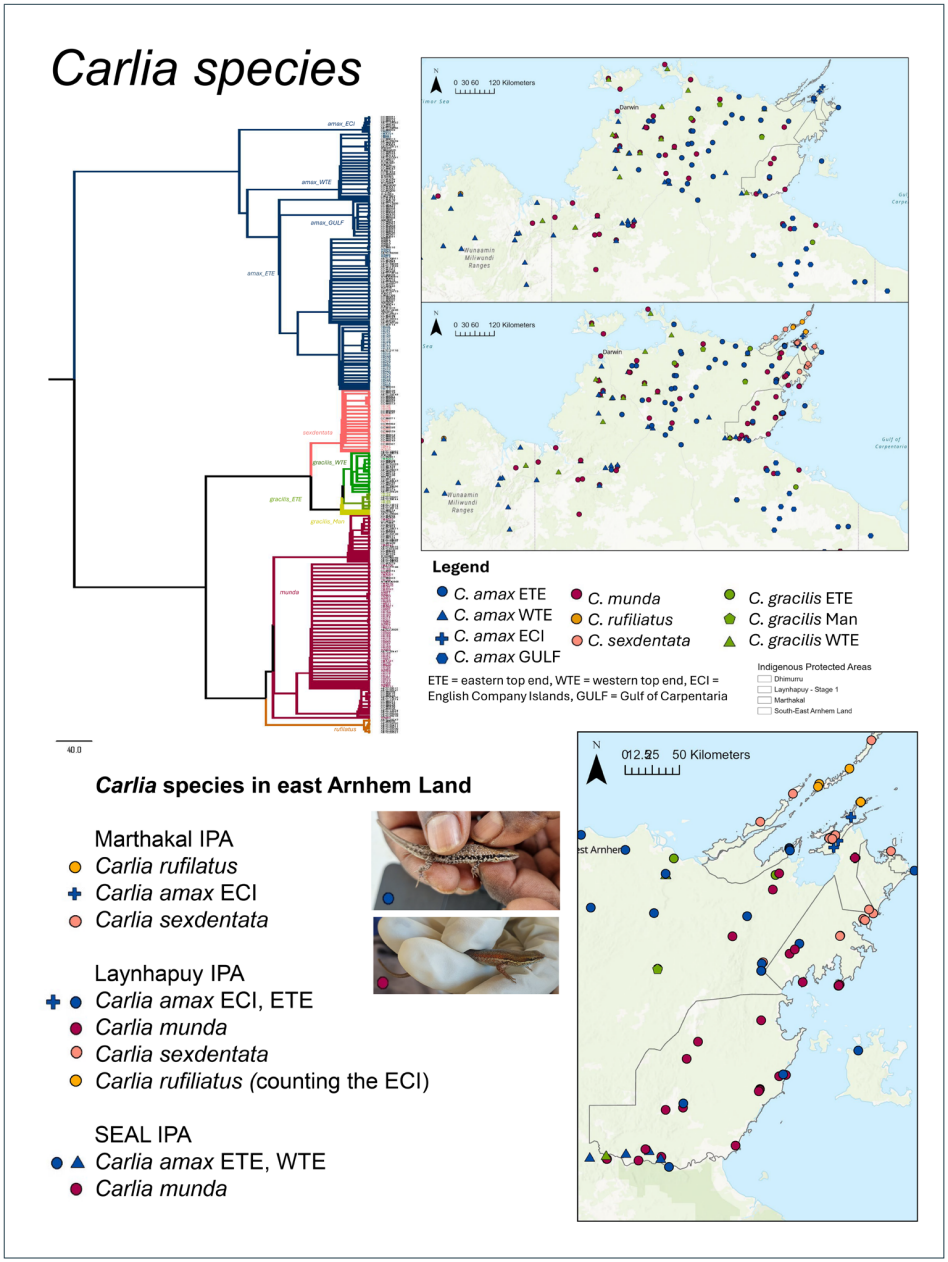
Example of the visual outputs used to discuss the genetic analysis results prepared by BC, here for *Carlia* species. These visual aids depict Western scientific ontology, using colour and symbols to indicate Western scientific classification of species and lineages of lizards across three Indigenous Protected Areas (IPA) (grey boundaries) in east Arnhem Land. A phylogenetic tree is displayed top left alongside species distribution maps (right), and a list of species and lineages within each IPA (bottom left).

Initial non‐structured and informal discussions between authors BC, BBW and BTM were held across two days at the Yirralka Ranger station in Gapuwiyak. These discussions were of varying lengths (10 mins—1 h, in between other work). In these sessions, the genetics results were presented by BC using visual aids (Figure 2) and discussed by BC, BBW and BTM. Mutual incomprehension was identified as the ontological divide between Yolŋu and Western scientific classifications of life; however, some complementary metaphors emerged. These initial sessions resulted in a more structured plan, suggested by BC and supported by BBW and BTM, to explore how the team could better contextualise the genetics results for Yolŋu. The team decided to embark on further discussions and negotiations of meanings (which later became identified as dhawurrpunarramirri) with Ŋaḻpaḻmi and other Rangers and create a video in Yolŋu Matha.

Thereafter, BBW, BTM and BC co‐researchers engaged in a more structured dhawurrpunarramirri session with each other (ca. 1–2 h) to further explore Yolŋu Matha metaphorical concepts that emerged from the initial discussions. The key metaphorical concepts were also discussed through structured dhawurrpunarramirri with other Yirralka Rangers (by BC, at a head ranger meeting in Baniyala) and Ŋaḻpaḻmi (by BBW, BTM and BC, in Gapuwiyak community) who had previously worked on the cross‐cultural fauna survey project. These metaphorical concepts were discussed (in English, or more predominantly Yolŋu Matha, led by BBW and BTM) following the initial presentation of the genetics results in English by BC (Figure 2). Due to time constraints (3–4 weeks of fieldwork by BC) of this project, and the need for this project to fit around other Yirralka Ranger djäma (work) and priorities, only a select group of Rangers and Ŋaḻapaḻmi that were involved in the cross‐cultural fauna survey project and had cultural authority to speak on the topic were approached (by BBW, BTM and BC) and available to engage in dhawurrpunarramirri. Dhawurrpunarramirri discussions with Rangers and Ŋaḻapaḻmi ranged from 20 min to 1.5 h.

Authors BBW, BTM and BC continued to engage in dhawurrpunaramirri discussions amongst themselves during the co‐creation of a video that sought to explain the genetics results to Yolŋu Matha speakers. These discussions ranged from 1 to 3 h and occurred over multiple weeks. Authors BBW and BTM then instructed BC to go over the Yolŋu Matha transcript for the video with MBW, a senior Ranger who had extensive professional Yolŋu Matha‐English translation and negotiation experience across a range of contexts, including complex legal situations. This resulted in further discussions and negotiations of meaning between Yolŋu and Western scientific ontologies. From this iterative process, we were able to find ‘common ground’ (Wunuŋmurra 1989) by drawing on Yolŋu metaphors as a form of negotiation to gain mutual comprehension about the following concepts: species, phylogenetic trees, speciation and phylogenetics. Author BC composed this article using notes and transcripts from these negotiations, under the guidance of authors FM and EE, clarifying details and the representation of meanings with BBW and BTM. We invite the reader to journey along with the authors as we reflect on our negotiations of mutual comprehension of Western scientific constructs and Yolŋu metaphors.

The Yolŋu knowledge featured in this paper remains the property of Yolŋu Ŋaḻapaḻmi. Knowledge shared was warraŋul (open) knowledge (see Wunuŋmurra 1989). A two‐stage free prior informed consent process was deployed in accordance with Macquarie University Human Research Ethics Committee approval (Ref: 5201800178): (1) consent before recording (audio and photos); and (2) consent to publish (quotes, photos) in research articles. Consent was provided in written form by each Ŋaḻapaḻmi and Ranger who engaged in dhawurrpunaramirri, through a prior and informed consent form. Each Ŋaḻapaḻmi was able to specify in these forms, through ticking boxes or a written description (transcribed from verbal direction) the extent to which their information, media (photos and audio) and knowledge would be shared.

### 5.1 Species

‘Species’ is a Euro‐Western concept, first used by Enlightenment era naturalists (from Latin: type) and refined by modern day scientists to meet a specific agenda: organising a ‘universal’ system for classifying life. The term species only became widely adopted in science following the binomial taxonomic system established by Linnaeus (Linnaeus 1735), and in general English language usage following Darwin's *On the Origin of Species* (Darwin 1859). The understanding of what constitutes a species has varied since the term's introduction, with the most commonly applied understanding today being the ‘biological species concept’ (Mayr 1942) defined by reproductive isolation. ‘Species’ can be considered a ‘human construction rather than an ontological reality’ and is not a term universally recognised or understood, particularly by Indigenous societies (Christie 1992). Its usage reflects a common ontological assumption in globally dominant science of discrete, objectively real units of nature—a tenet of the Western scientific worldview. It is not a term that is directly translatable in Yolŋu Matha; however, through dhawurrpunaramirri with Ŋaḻapaḻmi and the Yirralka Rangers, the research team identified three key terms that could be used to contextually ground the notion of ‘species’ for Yolŋu Matha speakers: miṯtji, mala and bäpurru (Appendix S1).

‘Species’ is not a taxonomic category used by Yolŋu. Rudder (1999) described the Yolŋu world as divided into two halves: walŋamirr (life having or living) and walŋamiriw (life without, or non‐living). He then suggested that there are nine main categories of living things that Yolŋu generally agree on (Rudder 1999). Amongst these, there is no discrete word for ‘animal’ (Rudder 1999), and no subordinate term that directly translates to ‘reptiles’. Furthermore, the Western species concept has no direct analogue in Yolŋu ontology or cosmology. Yolŋu do not classify animals based on reproductive isolation or fixed taxonomy; rather, their classifications can be considered relational, totemic and ecologically embedded (e.g., see Campbell et al. 2024; Yirralka Rangers, Macquarie University and Australian National University in preparation). As Christie (1992, 26) wrote, in Yolŋu Matha ‘there are very few names that divide the world up into macro‐categories English speakers imagine are really real’.

Yirralka Ranger PJ Ganbiḻpiḻ White spoke about miṯtji (lit: group (of people)) and how it *could* be used as a concept to discuss species, albeit not one used by Yolŋu.

Miṯtji, species, but we don't use that. We don't have a common and scientific species name. We just have a common name. Our scientific name would come from the totemic being of the actual fish. For example, common name ŋuykal' (Giant Trevally, *Caranx ignobilis* ), and scientific name gunuŋbal (Waŋarr (totemic) trevally), that's a more deeper name … only the totemic beings have that.

Here, PJ highlighted that ‘species’ is not a category that exists in Yolŋu Matha. However, miṯtji was discussed as a Yolŋu concept that could contextualise aspects of the species concept, although it operates within a different ontological frame. Miṯtji is a term used by Yolŋu to distinguish a distinct group of people that share similar qualities. It can be extended to ‘life forms’ more generally in this context. Nevertheless, PJ suggested Yolŋu do have ‘higher order’ or ‘deeper’ animal names, drawing parallels between Western scientific species names, and Yolŋu ‘deeper’ names that exist for Waŋarr (totemic beings). Like in Western science, Yolŋu have specialist, ‘deeper names’ for animals that they have a strong connection to. However, in contrast to the universalist ambition of Western scientific nomenclature, Yolŋu Waŋarr names are only used by those with cultural authority. Yolŋu nomenclature is based on relationality, and cultural connections, in contrast to the desire for separation and boundaries which underpins Western taxonomy. Yolŋu and Western scientific classifications are based on ‘incommensurable metaphysics’ that underpin the respective knowledge systems and go beyond fauna (e.g., see Verran (2002) on fire regimes).

Ŋaḻapaḻmi Muwarra Davis Marrawuŋgu highlighted the incommensurable metaphysics of Western scientific and Yolŋu ways of knowing animals. He revealed: ‘You describe that to us, the groups … because the whole [goal] of the animals naming in English … [is] putting through the species’. But for Yolŋu: ‘We have got to figure out how to connect with [the] species, [with] what type you are talking about … then we will go from there’. Here he communicated that knowing animals (alongside other walŋamirr and walŋamiriw beings) is dependent upon a relational understanding of connections with them. It is these connections that are essential to the understanding of an animal, the names it may have and who holds the cultural authority to share names and knowledge about it.

Whilst miṯtji refers specifically to a group of people, the word mala can be applied more broadly in Yolŋu Matha to mean a group consisting of multiple members of the same ‘type’ of being, or can be used as a marker of plurality (Charles Darwin University 2014). Because of this broader usage, mala was considered a more applicable term to relate to species by Ŋaḻapaḻmi through dhawurrpunaramirri negotiations. Muwarra Davis Marrawuŋgu (MDM) and Julie Yunupiŋu (JY) discussed this usage of the term mala:

MDM Like weṯi (wallabies) mala or guḏutjurrk (large two‐lined skinks) mala, whatever, yo [yes] species.

JY Like in a group, ga (and) group, ga group, like species. Mother nature nhakun (for example).

Mala can therefore be used by Yolŋu in a similar way that Balanda can use ‘species’: in the plural form to refer to genera or groups of different taxa, for example reptile species or *Carlia* species as reptiles mala or *Carlia* mala. However, mala is not a hierarchical classificatory term in the way that species or genus are in the Linnean taxonomic system. Consequently, a singular definition for the ‘biological species concept’ was harder to negotiate. The singular use encounters an ontological divide, where the incommensurability of Yolŋu and Western scientific knowledge systems and fundamentally different ways of understanding the universe meet. This ontological divide engendered mutual incomprehension. We also realised that the term ‘species’ is an example of an invariant noun; the spelling and pronunciation of both the singular and plural forms is identical. Thus, species can be applied as a concept defining the most basic singular classification of life or as a term that can refer to all life on Earth. This proved difficult to navigate across Western and Yolŋu knowledge and language systems. As Munurruŋ Bobby Wunuŋmurra commented, species was often understood in the plural form, to refer to all life:

Yo, species, mayali (meaning) is the whole. That's a scientific yäku (name), you know ‘species’. You can break it into smaller bits, that's mammal and reptile, meaning everybody, including us. Right? Am I right?

To overcome this mutual incomprehension around species, the term bäpurru (clan) was also offered by Yolŋu as a translational metaphor to help contextualise species. Since there are many different and distinct bäpurru in Yolŋu society, each known by a distinct name and shared ancestry, it was judged an appropriate term to refer to ‘wiripu'wiripu’ (different, distinct) types of animals, and as a form of classification different from animal groups recognised by Yolŋu. For example scientists recognise multiple different species within the Yolŋu Matha category of gunydjuḻu’ (small skinks). For Yolŋu, the extension of the term bäpurru from the human to the non‐human helps contextualise the Western differentiation into species using a Yolŋu framework (‘all humans are human, but they are also divided into bäpurru, while the same can be said of gunydjulu’).

Sharon Wunuŋmurra, translating for Marrarrawuy Margaret Waṉambi, elaborated as follows:

Barrkuwatj means it's like separate. So mayawa (Frilled Neck Lizard) is wangany (one), bäpurru wangany (one clan). But this one is [these are] all in one, but they are separate, separate bäpurru: gunydjuḻu, dhakarraŋbi, guḏutjurrk.

In effect, the Yolŋu animal category mayawa aligns with the Western nominal species, Frilled Neck Lizard ( *Chlamydosaurus kingii* ), and therefore can be said to ‘have one bäpurru’ (be one species). However, gunydjuḻu, guḏutjurrk and dhakarraŋbi have multiple bäpurru and therefore include many distinct groups, or rather species, from a Western perspective. Thus, the term can be used to help ground and contextualise Western scientific understanding using a Yolŋu concept. It applies a Yolŋu social category to refer metaphorically to a Western taxonomic concept, here species.

### 5.2 Phylogenetic Trees

Phylogenetic trees were the main Western genetic tool used to distinguish species of *Carlia, Ctenotus*, and *Diporiphora* lizards (Campbell et al. in review). The phylogenetic trees were also used to communicate the genetics results to Yolŋu, with one phylogenetic tree produced for each lizard genus and different species indicated by different coloured branches (Figure 2, Graphical Abstract). Yolŋu co‐researchers used two metaphors to contextualise their understanding of phylogenetic trees: gurruṯu and gurrkurr. Gurruṯu (kinship) is a key Yolŋu concept for describing connections between living things and comprises an underlying universal system of kin relations across east Arnhem Land (Blakeman and Burarrwaŋa 2023), similar to other intricate Indigenous kinship systems across Australia (Berndt and Berndt 1988). Gurrkurr describes a dimension of gurruṯu relating to the genealogical and ceremonial connections of and between bäpurru (clans; here, species) (Graphical Abstract).

When asked why the concept of gurruṯu could contextualise the phylogenetic trees, Sharon Wunuŋmurra translated for her ŋäṉḏi (mother), Marrarrawuy Margaret Waṉambi, that like Yolŋu ‘all animals have their own gurruṯu, they have their own kin connections’. However, gurruṯu proved to have two related but distinct uses. A distinction was drawn between animals that were considered part of Yolŋu gurruṯu, or as ‘gurruṯumirri’ (kinship having), including Waŋarr (totemic) animals such as garkman (green tree frog) (for Dhaḻwaŋu clan members), and those that were not part of Yolŋu gurruṯu, or not Waŋarr, but ‘ordinary’ animals like gunydjuḻu, which could be said to have ‘their own’ gurruṯu only amongst themselves.

With continued dhawurrpunaramirri negotiations, we found that gurruṯu did not align as well with the phylogenetic results as gurrkurr did. Gurrkurr is a ‘yindi yäku’ (lit: big name, powerful metaphor) for Yolŋu that characterises the metaphysical ancestral and ceremonial connections between Yolŋu as a root system, extending and branching out from the ‘trunk’ of the bäpurru. It is a multifaceted metaphorical concept drawn from the ‘natural’ world of which there are many examples in Yolŋu Matha (see Christie 1992; Morphy 2022; van Gelderen and Guthadjaka 2021). Whilst gurrkurr primarily means vein, it is metaphorically extended to ancestry (bloodline) and root systems (Charles Darwin University 2014) and also has connotations of ‘hidden’ beneath the skin (veins) (or the ground—roots) (discussed in Blakeman and Burarrwaŋa 2023). Hence it was generally thought a more apt metaphor for phylogenetic relationships which are ‘hidden’ in two senses. Firstly, gurrkurr is akin to a ‘scientific’ term that is ‘metaphorically hidden’ from the non‐scientist (or here, uninitiated in knowledge), and secondly, as with cryptic speciation, phylogenetic differences do not necessarily manifest themselves ‘on the surface’ in the form of easily observable physical differences. Additionally, gurrkurr is a tree‐related metaphor and the connection between roots and ancestry aligns well with the representation of common ancestry as the ‘root’ of the phylogenetic trees.

Whilst gurrkurr is usually applied in discussion of Yolŋu ancestry, Ŋaḻapaḻmi confirmed that gurrkurr was appropriate as a metaphorical concept that could be extended to the discussion of the phylogenetic trees generated for lizards. Gurrkurr was discussed as also resembling the Western metaphors of the ‘tree of life’ and ‘family tree’ that are similarly salient ‘non‐specialist’ terms for English speakers when conceptualising and discussing genealogical or DNA‐based connections.

As Julie Yunupiŋu described, ‘Like Yolŋu, they are branches, gurrkurr, like roots. Then it grows bigger and bigger and bigger. [Pointing to phylogenetic tree] They are like that. That DNA. It [gurrkurr] tells the story about DNA’.

Marrarrawuy Margaret Waṉambi (translated by Sharon Wunuŋmurra) concurred: ‘Gurrkurr, yeah, that's the line, connecting. Yo, manymak, that's the vein, gurrkurr, connecting to each other; joining the family…story of where you come from’.

Like the Balanda metaphor of a tree in phylogenetic ‘trees’, gurrkurr provides a model for the interconnections between related beings (in this case, lizards of the same and different species). However, for Yolŋu, gurrkurr extends beyond the geneological to encompass ceremonial ancestral connections. Blakeman and Burarrwaŋa (2023) described gurrkurr as representing connections; ‘ties of kinship’ defining mutual obligations and responsibilities, including, but expanding beyond (and not organised by) genetic closeness to include ceremonial relationships given to Yolŋu by Waŋarr (ancestral beings). Unlike the phylogenetic tree, gurkurr extends beyond genetic relations as a metaphor that invokes ceremonial connections. Thus it cannot be considered a direct translation but rather as a related concept that can be used to find common ground and contextualise Western scientific understanding and bridge ontologies.

### 5.3 Speciation and Phylogenetics

Mala'bunhamin, mala'barrkuwatj and mala'djarr'yun were identified by Yolŋu researchers as terms that could contextualise the Western scientfic concepts of speciation and phylogenetics.

**FIGURE 3:**
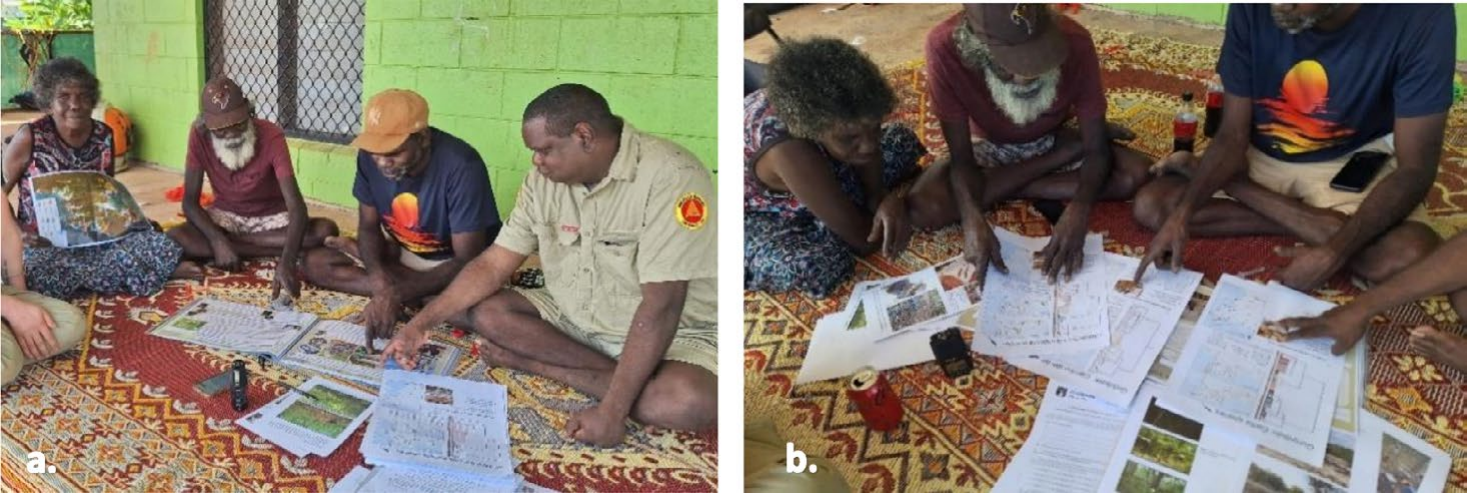
Co‐authors and Yirralka Rangers BBW and BTM in discussion with Gapuwiyak Ŋaḻapaḻmi Guthitjpuy Clancy Marrkula and Linda G. Bandawuŋu discussing: (a) the collection of lizard tissue samples during cross‐cultural fauna surveys; and (b) the results of the phylogenetic analyses.

Mala'bunhamin (from mala‐buma, lit: group‐create) translates as ‘to procreate’ or ‘giving new life to generations’, and mala'bunhamirr (reflexive form) ‘procreating together within distinct groups’ (Garŋgulkpuy and Marŋgithinyaraw 2002). These terms were identified by Yolŋu researchers and discussed with Ŋaḻapaḻmi in the context of speciation. These terms describe a process that connects the generations of the past, present and future within a ‘bloodline’ or across gurrkurr, as co‐author Butjiyaŋanybuy Thomas Marrkula (BTM) explained in dhawurrpunaramirri with his father and mother (Figure 3):

And every step, step ga (and) step. My father's father ga he found my dad, and dad found me, and I found my daughters, and daughters going to found my grandaughters. Mala'bunhamirr, that's [what] I'm saying mala'bunhamirri ga walal ga (they) guḏutjurrkdja (skinks).

As BTM explained and also noted in Garŋgulkpuy and Marŋgithinyaraw (2002), mala'bunhamirr generally refers to the continuous procreation of generations within a specific ‘family’ group in Yolŋu society. Here, he and his parents are suggesting it could be extended as a metaphor to explain the generational procreation of different bäpurru (here, species) of lizards. The definition of this term from a scientific perspective aligned well with the biological species concept, which defines species through reproductive isolation.

Mala'barrkuwatj (group‐become separate) was used to refer to the process of speciation in which lizards have separated into different groups over time. A variation of this term was used in Garŋgulkpuy and Marŋgithinyaraw (2002) to discuss how Yolŋu are ‘distributed into distinct groups’ and mala'barrkuwatjkunhawuy is translated as ‘(lit: groups constituted separately) distinct or differentiated’. It was also used in Blakeman and Burarrwaŋa (2023) to describe the way the gurrkurr of a clan spread out and become distinct. Thus again we arrived at a term that was used to describe Yolŋu social structure and interconnections, one that specifically related back to gurrkurr and can be metaphorically extended to animals to explain phylogenetic relationships.

‘Mala'djarr'yun’ (group choosing) was a third mala‐verb compound used to describe the process of speciation that was identified by Yolŋu researchers during dhawurrpunaramirri. ‘Mala'djarr'yun’ arose in two separate discussions with two different definitions in this context: (1) scientists ‘choosing what type’ of species each lizard was using genetics; and (2) the lizards themselves choosing how they would distribute themselves across Country (‘choosing Homelands’) where they would become their own type or bäpurru. The first description detailed understanding of the scientific methods and the outcome, and situated the human scientist as the actor, aligning with Western ontology. The second description demonstrated a perspective grounded in Yolŋu ontology in which the lizards are the actors (Campbell et al. 2024; Morphy 2022), here consciously forming groups, which vary across the landscape based on their choice of wäŋa (place, homeland), for which they are responsible (like Yolŋu) for looking after. Again, a concept used to describe Yolŋu social organisation could be metaphorically extended to lizards. As co‐author BBW explained to BC:

BBW Choosing the area, wäŋa. Same as that one, they moved from here and they are looking for this area, they are djarryun (choosing) this area. Djarryun. This is my area, I live here, choosing.

BC So they are deciding, the gunydjuḻu’?

BBW Yo, the gunydjuḻu’, guḏutjurrk, dhakarraŋbi. Mala'djarr'yunmirr (groups choosing having)– like you stay there and I stay that way and he gonna stay that way, and we are all family, and we have to look after our land. Makes sense?

### 5.4 Non‐Translatable Words: Recognising Ontological Incommensurability

**FIGURE 4:**
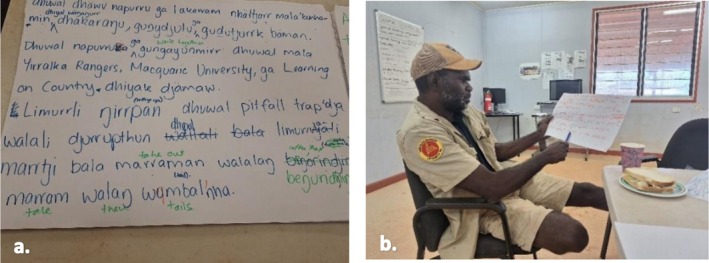
Photographs of the transcription process for key genetic results: (a) Yolŋu Matha transcription; and (b) author BTM checking spelling, grammar and meaning.

Certain Western scientific terms like ‘scientist’ and ‘sample’ were recognised by Yolŋu speakers, and no translation was really considered necessary. The border of epistemic limits was drawn for these words that were considered to have ‘bäyŋu dhäruk Yolŋu mala’, no direct or metaphorical translation for Yolŋu Matha speakers, like ‘sequence’ and ‘DNA’. Previous attempts to translate DNA, including ‘guwayak warrakan’ (lit: wrist animal) (PJ White pers. comm) and ‘djinga'puy wäyuk’ (lit: underneath place arm) (ARDS Rumbalpuy Dhäwu App), were brought up in discussion by BC, the Balanda researcher. These terms were not recognised as salient terms by Ŋaḻapaḻmi or Yolŋu researchers. Yolŋu researchers and Ŋaḻapaḻmi decided that the Balanda words for these terms would be used in the genetics results communications. This recognised the incommensurability of some Yolŋu and Western scientific concepts as discussed by authors MBW and BC below when reviewing the Yolŋu Matha transcription for the planned video (Figure 4):

BC Will everyone know what this word ‘sequence’ means?

MBW Yaka, but it's hard to explain; I would need five or six paragraphs to explain it. So we will just leave the Balanda word.

BC Should we say it's (sequence) like a written dhäwu (story)?

MBW Yo, we can put it somewhere, but where can we put it?

Here, … DNAdja dhu sequenced, mayali mirriyaman ga dhäwu mirriyum malŋ'maraman wanhaŋur gunydjuḻu’ mala'bunhamin. After they find the DNA, the DNA (sequence) is (a) meaningful story and (scientists) make it into reality where that gunydjuḻu’ was genetically originated from, that's what it says up here.

That's it, Yolŋu Matha and Balanda Matha has similar understanding now.

## 6 Reflection: Dhawurrpunaramirri and the Usefulness of Metaphors

In the present study, mutual incomprehension was bridged by applying an Indigenous research methodsology; Yolŋu dhawurrpunaramirri, to discuss and negotiate culturally salient metaphors that contextualised and helped explain Western science genetics results. Dhawurrpunaramirri allowed the Yolŋu and Balanda research team to find conceptual common ground and thus mutual comprehension of Western scientific (and through the process, for the Balanda, of Yolŋu Matha) terms. The Yolŋu Yirralka Ranger co‐researchers and Ŋaḻapaḻmi recommended metaphors and terms typically used to conceptualise Yolŋu social structure to contextualise the wildlife genetics results. These concepts are thus in no way direct translations but terms nominated by Yolŋu researchers and Ŋaḻpaḻmi as appropriate metaphors to negotiate a shared, culturally relevant ‘common ground’ of mutual understanding. Whilst gurrkurr was offered as a metaphor that aligned well with tree (in phylogenetic tree), other terms miṯtji, mala, bäpurru, mala'bunhamin, mala'barrkuwatj and mala'djarr'yun were not as ‘naturally’ invoked. They became metaphors in this context by their extension beyond Yolŋu social structure to explain hereditary connections and distinctiveness in small lizards (a level of taxonomy not traditionally recognised by Yolŋu). In other words, Yolŋu social organisation (including clan‐based and ceremonial connections) emerged as an overarching metaphor (grounded by the more tangible metaphorical connection of gurrkurr), enabling us, Yolŋu and Balanda researchers, to find conceptual common ground.

Bridging the philosophical and linguistic space between two worldviews and languages was a complex task, and there are limitations to the extension of metaphors. Reid and Rout (2016, 429) defined Indigenous relationality, an underlying value in Indigenous research methodologies as: ‘reciprocal and contextual rather than unidirectional and abstract, and that as these relationships progress each entity shapes the other in meaningful ways’. The same can be said for the professional relationships that developed between Yolŋu and Balanda on this research team, and the mutual understandings generated. In the same way that the Yolŋu Yirralka Ranger co‐researchers and Ŋaḻapaḻmi have not necessarily walked away with technical knowledge of DNA sequencing and phylogenetic tree construction, so too the Balanda university researchers have not walked away with deep, technical knowledge of Yolŋu social organisation and metaphysics. As author MBW remarked, ‘That's it, Yolŋu Matha and Balanda Matha have similar understanding now’. What we have done is negotiated a common ground, where we have been able to understand each other better in line with local Yolŋu and international aspirations for equitable and ethical knowledge sharing (Marika et al. 2009; Smith 2012; Wunuŋmurra 1989). By engaging with dhawurrpunaramirri we were able to ‘interrogate the differences and similarities between the “two” systems of knowledge’ (Morphy 2003, 8) and generate the ability to apply new or existing concepts to negotiate cross‐cultural understanding for future contexts.

Metaphors have long been central to both science communication and Yolŋu knowledge sharing, and there is evidence to suggest they are a universally important linguistic translation tool (Lakoff and Johnson 1980; Morphy 2022; Rout and Reid 2020). However, as languages describe the world in different ways, metaphors are not always salient across cultures (Morphy 2022; Morphy and Morphy 2020). Western science communication hinges on metaphors that cannot always be considered objective or universal. Assuming that English metaphors are understood universally can undermine the impact of science and science communication in cross‐cultural contexts, including in the conservation space. However, we demonstrated that purpose‐specific identification of culturally salient metaphors and concepts can support equitable knowledge sharing in cross‐cultural research projects. We demonstrated that engagement with Yolŋu dhawurrpunaramirr both highlighted and remedied mutual incomprehension in a culturally grounded way that centered Indigenous onto‐epistemologies and authority, bringing Indigenous ways of understanding and relating to the world to the fore. Through application of locally preferred Indigenous research methodologies, Western scientists can support the decolonization of science by supporting the capacity of Indigenous research partners to exercise self‐determination and promote their knowledge systems in conservation research (Muller 2014), whereas concurrently building ‘flexibility to live in both worlds’ (Wunuŋmurra 1989, 12).

## Author Contributions

**Bridget Campbell:** conceptualization (equal), data curation (lead), funding acquisition (equal), investigation (equal), methodology (equal), writing – original draft (lead), writing – review and editing (equal). **Banygada Brendan Wunuŋmurra:** conceptualization (equal), investigation (equal), methodology (lead), validation (equal). **Butjiyaŋanybuy Thomas Marrkula:** conceptualization (equal), investigation (equal), methodology (lead), validation (equal). **Munurruŋ Bobby Wunuŋmurra:** conceptualization (supporting), investigation (supporting), methodology (supporting), validation (equal). **Yirralka Rangers:** conceptualization (equal), funding acquisition (equal), investigation (equal), methodology (equal), resources (equal), validation (equal). **Frances Morphy:** conceptualization (equal), funding acquisition (equal), methodology (supporting), supervision (equal), validation (equal), writing – review and editing (equal). **Emilie Ens:** conceptualization (supporting), funding acquisition (equal), supervision (lead), writing – review and editing (equal).

## Ethics Statement

This project was approved by the Macquarie University Human Research Ethics Committee (Reference No: 520241637155816).

## Conflicts of Interest

The authors declare no conflicts of interest.

## Supporting information


**Appendix S1:** ece372274‐sup‐0001‐Supinfo.docx.

## Data Availability

Aligning with the CARE principles and Indigenous Data Sovereignty and Governance protocols, Yolŋu knowledge holders remain the owners of knowledge shared during this project. Dhawurrpunaramirri transcripts were made available via the Open Science Framework for the review process. Following publication, transcripts will be made available granted permission of Indigenous data custodians. The providence of these transcripts and knowledge shared in them is marked by the Local Contexts Hub Biocultural (BC) and Traditional Knowledge (TK) notices. Local Contexts Hub Project ID: aea66340‐4843‐4b52‐8229‐d909977abe02. Access can be requested via the Open Science Framework platform: https://doi.org/10.17605/OSF.IO/G3XYE.
