# Supplementary material for: Finding Common Ground: Indigenous Research Methods Facilitate Scientific Knowledge Sharing in Cross‐Cultural Wildlife Research
Source: Ecol Evol. 2025 Oct 10;15(10):e72274. doi: 10.1002/ece3.72274 (PMC12513723; doi:10.1002/ece3.72274)
Supplement: Supplementary file 1 — Appendix S1: ece372274‐sup‐0001‐Supinfo.docx. [file ECE3-15-e72274-s001.docx]

**Appendix 1.**

Yolŋu Matha (predominantly Djambarrpuyŋu dialect) terms used in this article with English translations and metaphorical extensions discussed here or elsewhere.

| **Yolŋu Matha term** | **English translation** | **Metaphorical extensions** |
| --- | --- | --- |
| Balanda | Non-Indigenous people, generally Euro-Australian people. |  |
| bala ga lili | give and take | Reciprocal intercultural relationships (see |
| bäpurru | clan – Yolŋu social group constituted through patrilineal descent | Here: taxonomic classification within Yolŋu identified classification of animals, i.e. Western identified species. |
| barrkuwatj | separate |  |
| bäyŋu | none |  |
| Dhakarraŋbi | Small dragons (including *Diporiphora spp.)* |  |
| Dhaḻwaŋu | Name of clan |  |
| dhäruk | terminology, language |  |
| dhäwu | story, narrative |  |
| dhawurrpunaramirri | interrupting each other | Both-ways discussion and negotiation between Yolŋu and Balanda (see Wunuŋmurra 1989).  Here: as an Indigenous Research Method (IRM) to work towards mutual comprehension in cross-cultural ecology research. |
| dhu | future marker |  |
| Dhuwa | moiety name |  |
| -dja | focus suffix |  |
| djarryun | Choosing, deciding |  |
| djinga’puy wäyuk | Lit: underneath place arm | Previously recorded as translation for DNA by ARDS (2019). |
| ga | and |  |
| Garkman | Green tree frog (*Litoria caerulea*) |  |
| Guḏutjurrk | Large two-lined skinks (including *Ctenotus* spp.) |  |
| Gunuŋbal | Waŋarr giant trevally |  |
| Gunydjuḻu | Small skinks (including *Carlia* spp.) |  |
| gurrkurr | Venous system | By extension: root system and ‘blood line.’ (E.g. see Blakeman and Burarrwaŋa, 2023)  Here: genetic ancestry; DNA. |
| gurruṯu | Yolŋu kinship |  |
| gurruṯumirri | kin(ship) having |  |
| guwayak warrakan | Lit: wrist animal (noted as previous translation of DNA, pers. comms. PJ White). |  |
| mala | group | Here: taxonomic classification within Yolŋu identified classification of animals, i.e. Western identified species. |
| mala’barrkuwatj | group-become separate | Here: speciation |
| mala’bunhamin | from *mala-buma*, lit: group-create, procreate | Here: speciation |
| mala’bunhamirr | (Reflexive form of mala’bunhamin) ‘procreating together within distinct groups’ (Garŋgulkpuy and Marŋgithinyaraw, 2002) | Here: speciation |
| mala’djarr’yun’ | group-choosing | Here: speciation |
| maḻŋ’maraman | searching for |  |
| manymak | good |  |
| mayali | meaning |  |
| Mayawa | Frilled Neck Lizard, *Chlamydosaurus kingii* |  |
| mirriyaman | meaning that |  |
| miṯtji | lit: group (of people) |  |
| nhakun | for example |  |
| ŋaḻapaḻmi | Elders; senior Yolŋu knowledge holders |  |
| ŋäṉḏi | Mother |  |
| Ŋuykal’ | King Fish, Giant Trevally, *Caranx ignobilis* |  |
| walal | they, |  |
| walŋamiriw | life-without, non-living |  |
| walŋamirr | life-having, living |  |
| waŋgany | one |  |
| wanhaŋur | Where from |  |
| wäŋa | home, place, habitat |  |
| wäŋa waṯaŋu | Land holder, Traditional Owner, |  |
| Waŋarr | Ancestral beings, totemic beings/entities |  |
| warrakan | meat, by extension animal (although does not align completely with English/scientific classification of animal) |  |
| Weṯi | Wallaby |  |
| wiripu’wiripu | different, distinct |  |
| yaka | no |  |
| yäku | name |  |
| yindi | big |  |
| yindi yäku | lit: big name | Powerful metaphor |
| Yirritja | moiety name |  |
| yo | yes |  |
| Yolŋu | Aboriginal person from east Arnhem Land; person |  |
| Yolŋu Matha | Yolŋu Language |  |

**References**

Aboriginal Resource Development Services (ARDS). 2019. Rumbalpuy Dhäwu (Mobile Application). Downloaded from: <https://play.google.com/store/apps/details?id=au.com.bock.ards&hl=en_AU&pli=1>.

Blakeman, B., Burarrwaŋa, D., 2023. Yolkala Gumurrlili? with Whom Towards the Chest? A Relational Portrait of Yolŋu Social Organisation. Journal of Intercultural Studies 44, 678–696. https://doi.org/10.1080/07256868.2023.2198202

Garŋgulkpuy, J., Marŋgithinyaraw, Y., 2002. Yolŋu Baḻandi-waṯaŋumirr: Yolŋu with ancestral connections. Centre for Indigenous Natural and Cultural Resource Managament, Faculty of Indigenous Research and Education, Northern Territory University. Accessed from: <https://digitalcollections.cdu.edu.au/nodes/view/7849>. (Accessed 30/8/2024).

Wunuŋmurra, W., 1989. Dhawurrpunaramirri: finding common ground for a new Aboriginal curriculum. Ngoonjook, Batchelor Journal of Aboriginal Education 12–16.
